# A Clinical Study of Thyroid Abnormalities and Autoantibodies in Patients Diagnosed With Anti-nuclear Antibody Positive Autoimmune Connective Tissue Disorders: An Observational Cross-Sectional Study

**DOI:** 10.7759/cureus.65950

**Published:** 2024-08-01

**Authors:** Saimounika Adapa, Varsha R Bhatt, Govind Shiddapur, Nilesh Jagdale, Vutukuru Kalyan Kumar Reddy, Mohith Prakash Kondapalli, Sonali Agarwal, Diksha Sabharwal, Advait Jha

**Affiliations:** 1 Department of General Medicine, Dr. D. Y. Patil College, Hospital and Research Centre, Dr. D. Y. Patil Vidyapeeth, Pune, IND; 2 Department of Clinical Immunology and Rheumatology, Bharati Vidyapeeth (Deemed to be University) Medical College and Hospital, Pune, IND

**Keywords:** thyroid abnormaliities, subclinical hypothyroidism (such), thyroid function tests (tfts), anti-nuclear antibody (ana), autoimmune connective tissue disorders (ctds)

## Abstract

Introduction: Autoimmune connective tissue disorders (CTDs) are characterized by inflammation of the connective tissue structures and immune system aberrations, such as autoantibody production. This study investigates the prevalence and clinical significance of thyroid abnormalities in patients with anti-nuclear antibody (ANA)-positive autoimmune CTDs.

Methods: This prospective cross-sectional observational study was conducted at Dr. D. Y. Patil Medical College, Hospital and Research Centre, Dr. D. Y. Patil Vidyapeeth (Deemed to be University), Pune, from September 2022 to June 2024. Eighty patients diagnosed with ANA-positive CTDs were included. Comprehensive histories were collected from them and clinical examinations and routine investigations were performed. Blood samples were collected for thyroid function tests and autoantibody tests. Thyroid ultrasound investigations were also performed. Ethical approval and informed consent were obtained.

Results: The study revealed a significant prevalence of thyroid dysfunction among participants, with 39 (48.75%) exhibiting some form of thyroid abnormality. Subclinical hypothyroidism was the most common condition in 18 (22.50%) participants, predominantly affecting females. Thyroid autoantibodies were present in 32 (40%) participants, with thyroid peroxidase antibodies (anti-TPO Ab) being the most common seen in 17 (21.25%) participants. Systemic lupus erythematosus (SLE) was the most prevalent CTD among participants, seen in 44 (55%) participants, followed by Sjogren’s syndrome (SS) seen in 19 (23.75%) participants.

Conclusion: The study underscores the necessity of routine thyroid function screening in patients with ANA-positive CTDs to facilitate early detection and management of thyroid abnormalities, thereby preventing progression to overt hypothyroidism or hyperthyroidism. The findings highlight the significant association between thyroid dysfunction and autoimmune CTDs, advocating for a holistic approach to patient care.

## Introduction

Autoimmune connective tissue disorders (CTDs) encompass a diverse array of conditions characterized by inflammation in connective tissue-rich structures like skin and joints, coupled with immunoregulatory aberrations such as autoantibody production and disruptions in cell-mediated immunity [1]. Systemic autoimmune CTDs, constituting a subset of these conditions, involve the body's immune system mistakenly attacking its own tissues, often without a known cause, resulting in diseases like rheumatoid arthritis (RA), systemic lupus erythematosus (SLE), polymyositis, dermatomyositis, systemic sclerosis (SSc), Sjogren’s syndrome (SS), mixed connective tissue disease (MCTD), undifferentiated connective tissue disorder (UCTD), and overlap syndromes [2].

Thyroid diseases, particularly autoimmune thyroid disorders like Hashimoto’s thyroiditis and Graves’ disease, have been associated with CTDs. The prevalence of thyroid abnormalities, including hypothyroidism, hyperthyroidism, and autoimmune thyroid diseases, is higher in patients with CTDs compared to the general population, suggesting a potential link between these conditions [3]. While the precise mechanisms underlying this association remain unclear, genetic predisposition, environmental factors, immune dysregulation, and hormonal changes are thought to contribute to the development of poly-autoimmunity [4].

Regional disparities exist in the prevalence of thyroid disorders among individuals with autoimmune CTDs, with higher rates reported in certain regions such as India and Brazil. Understanding this association is crucial, as undiagnosed thyroid abnormalities in CTD patients can exacerbate their existing conditions and affect their quality of life [5]. Moreover, overlapping symptoms between thyroid disorders and CTDs may lead to underdiagnosis of thyroid disease in CTD patients, necessitating heightened awareness and comprehensive screening protocols [6].

The diagnosis of autoimmune thyroid disease is often supported by the presence of specific autoantibodies targeting thyroid antigens, such as anti-thyroid peroxidase antibodies (anti-TPO Ab), thyroglobulin antibodies (anti-Tg Ab), and thyroid-stimulating hormone receptor antibodies (TSH Rs Ab). These antibodies play a diagnostic role in conditions like Hashimoto’s thyroiditis and Graves' disease, aiding in the differentiation and management of thyroid disorders in CTD patients [7]. While thyroid abnormalities have been extensively studied in certain CTDs like SLE, RA, SSc, and SS, literature is scarce on this subject in specific regions. Hence, this study aims to fill this knowledge gap by evaluating thyroid function tests, thyroid antibodies, and abnormalities in anti-nuclear antibody (ANA)-positive CTD patients, shedding light on the prevalence and clinical significance of thyroid disorders in this population.

Aims and objectives

The study aims to investigate thyroid abnormalities, thyroid function tests, and autoantibodies in patients with ANA-positive autoimmune connective tissue disorders by determining the prevalence of thyroid autoantibodies and examining the association between abnormal thyroid function tests and thyroid autoantibodies in these patients.

## Materials and methods

Study setting and design

This prospective, cross-sectional observational study was conducted at a 2011-bedded tertiary care center, Dr. D. Y. Patil Medical College, Hospital and Research Centre, Dr. D. Y. Patil Vidyapeeth (Deemed to be University), Pune, from September 2022 to June 2024. Eighty cases diagnosed with ANA-positive autoimmune CTDs based on the latest diagnostic criteria were included in the study. Written informed consent in the patients' vernacular language was obtained before the study commenced.

Inclusion criteria

Patients aged 16 years and older, both males and females, diagnosed with ANA-positive autoimmune CTDs by immunofluorescence (IF) technique b (titer > 1:100), including SLE-based on Systemic Lupus International Collaborating Clinics (SLICC) criteria, SSc based on ACR/EULAR (American College of Rheumatology/ European League Against Rheumatism) criteria, SS according to ACR/EULAR criteria, UCTD, MCTD diagnosed with Kasukawa criteria, and polymyositis/dermatomyositis diagnosed with Bohan and Peter criteria, were included in the study.

Exclusion criteria

Patients previously diagnosed with thyroid abnormalities and on medication, those with sepsis or active infections, patients currently undergoing high-dose steroid therapy (500 to 1000 mg methylprednisolone per day), those with a history of thyroid radiation exposure or surgery, those who have received chemotherapy for malignancy, and those with chronic liver disease, chronic kidney disease, HIV infections, or on medications such as antipsychotics, Amiodarone, interleukins, and tyrosine kinase inhibitors, as well as pregnant women, were excluded from the study.

Sample size

Considering the prevalence of thyroid dysfunction in patients diagnosed with autoimmune CTDs to be 41% according to Jones et al. [5], at an acceptable difference of 11%, a 95% confidence interval, and a power of 80%, the minimum sample size was calculated to be 80. The software used is WinPepi V.11.65.

Data and sample collection

Eighty patients who met the inclusion criteria were enrolled in the study. Comprehensive histories were taken, covering symptoms like cold and heat intolerance, tremors, palpitations, weight changes, menstrual abnormalities, and other thyroid-related symptoms. Thorough clinical examinations, including detailed thyroid assessments, were conducted. Routine investigations included a complete blood count (CBC), liver function tests (LFTs), renal function tests (RFTs), and assessing levels of serum electrolytes, serum proteins, erythrocyte sedimentation rate (ESR), and C-reactive protein (CRP). ANA-IF (anti-nuclear antibody-immunofluorescence) and ANA blots were recorded for all the cases. Blood samples were collected after an eight-hour fast for thyroid function tests (T3, T4, and TSH) and anti-TPO antibody tests. Anti-Tg and TSH receptor antibodies were also sent to all the patients.

Consent

Participants were thoroughly informed about the study's aims, procedures, risks, and benefits in their native language, and written informed consent was obtained before inclusion. This process ensured ethical integrity and participant autonomy throughout the study.

Statistical analysis

In our study, categorical variables were presented as frequency and percentages, and quantitative variables were expressed as mean ± SD at a 95% confidence interval. Data tracking was performed using Microsoft Excel (Microsoft Corporation, Redmond, WA, USA) and analyzed using Jamovi version 2.4.12.0 and Epi Info 7.

## Results

Our study of 80 participants diagnosed with ANA-positive autoimmune CTDs revealed a significant gender disparity, with 65 (81.25%) females and 15 (18.75%) males (Table 1). It also showed that the largest age group comprised individuals aged 40 years or younger (n=43, 53.75%), followed by those aged 41-50 years (n=23, 28.75%), and 51-70 years (n=14, 17.5%). The overall mean age was 46.76 ± 11.32 years, ranging from 19 to 70 years (Table 2).

**Table 1:** Sex-wise distribution of the study participants n: number; %: percentage

Sex	n (%)
Male	15 (18.75%)
Female	65 (81.25%)
Total	80 (100%)

**Table 2:** Age-wise distribution of the study participants SD: standard deviation

Age in years	Number	Percentage (%)	Mean ± SD
≤ 40	43	53.75	46.76 ± 11.32
41-50	23	28.75	
51-70	14	17.5	
Total	80	100	

In our study, 41 (51.25%) participants had normal thyroid function, while 39 (48.75%) exhibited thyroid dysfunction (subclinical hypothyroidism, hypothyroidism, and hyperthyroidism). Thyroid dysfunction was more prevalent among females. Females predominantly exhibited subclinical hypothyroidism (n=18, 22.50%) and hypothyroidism (n=17, 21.25%). Hyperthyroidism was present in four (5%) of the participants, all of whom were female, with no cases of subclinical hyperthyroidism detected (Table 3).

**Table 3:** Prevalence of Thyroid dysfunction among study participants n: number; %: percentage

Thyroid dysfunction	Male, n (%)	Female, n (%)	Total, n (%)
Normal	7 (8.75%)	34 (42.5%)	41 (51.25%)
Subclinical hypothyroidism	4 (5%)	14 (17.5%)	18 (22.5%)
Hypothyroidism	4 (5%)	13 (16.25%)	17 (21.25%)
Subclinical hyperthyroidism	0	0	0
Hyperthyroidism	0	4 (5%)	4 (5%)
Total	15 (18.75%)	65 (81.25%)	80 (100%)

Among the 80 participants, 32 (40%) tested positive for thyroid autoantibodies. Specifically, anti-TPO antibodies were present in 17 (21.25%) participants, with the highest occurrence in those with hypothyroidism, seen in nine (11.25%) participants. TSH receptor antibodies were found in eight (10%) participants, primarily in those with hypothyroidism, as seen in five (6.25%) participants. Anti-Tg antibodies were detected in seven (8.75%) participants, notably in those having hypothyroidism (n=3, 3.75%) and hyperthyroidism (n=2, 2.5%). Thyroid autoantibodies were not present in participants with normal thyroid function or subclinical hyperthyroidism (Table 4).

**Table 4:** Prevalence of thyroid autoantibodies and their association with thyroid dysfunction among study participants Anti-Tg antibodies, anti-thyroglobulin antibodies TSH receptor antibodies, thyroid-stimulating hormone receptor antibodies anti-TPO antibodies, anti-thyroid peroxidase antibodies %: Percentage; n: number

Thyroid dysfunction	Anti-TPO antibodies, n (%)	TSH receptor antibodies, n (%)	Anti-Tg antibodies, n (%)	Total antibody positive, n (%)
Normal	0 (0%)	0 (0%)	0 (0%)	0 (0%)
Subclinical hypothyroidism	5 (6.25%)	3 (3.75%)	2 (2.5%)	10 (12.5%)
Hypothyroidism	9 (11.25%)	5 (6.25%)	3 (3.75%)	17 (21.25%)
Subclinical hyperthyroidism	0 (0%)	0 (0%)	0 (0%)	0 (0%)
Hyperthyroidism	3 (3.75%)	0 (0%)	2 (2.5%)	5 (6.25%)
Total	17 (21.25%)	8 (10%)	7 (8.75%)	32 (40%)

According to the study, SLE was the most prevalent connective tissue disorder among the participants, affecting 44 (55%), all of whom were female. SS was the second most common, affecting 19 (23.75%) participants, out of which six (7.5%) were male and 13 (16.25%) female. Myositis was present in nine (11.25%) participants, while UCTD, SSc, and MCTD were less common, affecting five (6.25%), two (2.5%), and one (1.25%) participants, respectively. Regarding thyroid dysfunction, SLE had the highest prevalence among those with thyroid abnormalities, with 19 (23.75%) participants with SLE exhibiting thyroid dysfunction, followed by 12 (15%) participants with SS. Myositis showed a prevalence in four (5%) participants having thyroid dysfunction, while UCTD, SSc, and MCTD had lower rates of thyroid dysfunction. The prevalence of thyroid autoantibodies among the participants with connective tissue disorders was highest for anti-TPO antibodies (n=12, 15%) in SLE patients, while TSH receptor antibodies were found in eight (10%) participants, occurring predominantly in five (6.25%) patients with SLE and in two (2.5%) patients with myositis. Anti-Tg antibodies were observed in seven (8.75%) participants, mainly in four (5%) SLE patients (Table 5).

**Table 5:** Distribution of connective tissue disorders and thyroid autoantibodies among the study participants Anti-Tg Antibodies: Anti-thyroglobulin antibodies TSH Rs antibodies: Thyroid-stimulating hormone receptor antibodies Anti-TPO Antibodies: Anti-thyroid peroxidase antibodies MCTD: Mixed connective tissue disease; UCTD: undifferentiated connective tissue disorder; SLE: systemic lupus erythematosus %: Percentage; n: number

Connective tissue disorder	Male, n (%)	Female, n (%)	Total, n (%)	Subclinical hypothyroidism, n (%)	Hypothyroidism, n (%)	Hyperthyroidism, n (%)	Anti-TPO antibodies present, n(%)	TSH Rs antibodies present, n(%)	Anti-Tg antibodies present, n(%)
Sjogren's syndrome	6 (7.5%)	13(16.25%)	19(23.75%)	4 (5%)	8 (10%)	0	3 (3.75%)	1 (1.25%)	0
SLE	0	44 (55%)	44 (55%)	9 (11.25%)	7 (8.75%)	3 (3.75%)	12 (15%)	5 (6.25%)	4 (5%)
UCTD	3(3.75%)	2 (2.5%)	5(6.25%)	1 (1.25%)	0	1 (1.25%)	0	0	1 (1.25%)
Systemic Sclerosis	1(1.25%)	1 (1.25%)	2 (2.5%)	0	1 (1.25%)	0	1 (1.25%)	0	1 (1.25%)
MCTD	0	1 (1.25%)	1(1.25%)	1 (1.25%)	0	0	0	0	0
Myositis	5(6.25%)	4 (5%)	9(11.25%)	3 (3.75%)	1 (1.25%)	0	1 (1.25%)	2 (2.5%)	1 (1.25%)
Total	15(18.75%)	65(81.25%)	80(100%)	18 (22.50%)	17 (21.25%)	4 (5%)	17 (21.25%)	8 (10%)	7 (8.75%)

## Discussion

This study focused on a cohort of 80 participants diagnosed with ANA-positive CTD. The demographic characteristics of the study population showed a predominance of females, accounting for 65 (81.25%) participants. This finding is consistent with various studies indicating a higher prevalence of autoimmune disorders among women, as exemplified by the works of Jaring et al. [8]. and Arnaout et al. [9]. Jaring et al. [8] observed a prevalence of autoimmune disorders among 98.71% of the women in a study of 155 patients, while Arnaout et al. [9] reported the prevalence of these disorders in 69.4% of the female participants in their cohort of 118 individuals. The mean age of participants in our study was 46.76 ± 11.32 years, aligning closely with the previous findings, such as Arnaout et al.'s [9] reported mean age of 34.4 ± 1.4 years.

Our study revealed a significant prevalence of thyroid dysfunction among participants, with 39 (48.75%) showing some form of thyroid abnormality. Subclinical hypothyroidism was the most common condition, affecting 18 (22.5%) participants. Shaheen Akhtar et al. [10] found that 19 (9.5%) of the 200 patients in their study had subclinical hypothyroidism, 16 (8%) had clinical hypothyroidism, and 12 (6%) had hyperthyroidism. The high prevalence of thyroid dysfunction in our cohort underscores the importance of regular screening for thyroid abnormalities in patients with autoimmune CTDs.

SLE was the most prevalent autoimmune condition among our participants, affecting 44 (55%) of the cohort, followed by SS seen in 19 (23.75%) patients. These findings are in line with the existing literature, which frequently reports a high incidence of SLE among patients with autoimmune CTDs. According to Rahman et al. [11], SLE is one of the most common autoimmune diseases, particularly among females, a trend that our study mirrored, with the majority of SLE and SS cases being female. The observation of myositis in 11.25% of the participants underscores the importance of comprehensive screening for multiple autoimmune conditions, given their frequent coexistence and shared pathophysiological mechanisms [11].

Thyroid dysfunction was particularly prevalent among SLE and SS patients, with 19 (23.75%) and 12 (15%) participants, respectively, exhibiting thyroid abnormalities. These results are similar to those of Franco and Amaya-Amaya J [12], who found that people with SLE and SS often have problems with their thyroids because both the conditions are caused by the immune system. Additionally, a notable prevalence of thyroid disorders in myositis patients was found in their study, a trend also observed in our study with a prevalence of thyroid dysfunction in four (5%) myositis patients.

In addition, 32 (40%) patients who participated in our study had thyroid autoantibodies. The most common ones were anti-TPO antibodies seen in 17 (21.25%) participants, followed by TSH receptor antibodies seen in eight (10%) participants, and finally anti-Tg antibodies seen in seven (8.75%) participants. The high prevalence of these antibodies highlights a strong association between thyroid dysfunction and autoimmune CTDs. Anti-TPO antibodies were found in a significant number of people who had hypothyroidism, which supports the idea that these antibodies are linked to thyroid problems in autoimmune conditions. Ye An Kim et al. [13] reported that subclinical hypothyroidism and positivity for antithyroid antibodies were about twice as high in females, potentially explaining the higher prevalence of autoimmune thyroid diseases in women. Fatourechi et al. [14] also found that patients with subclinical hypothyroidism have a high rate of progression to clinically overt hypothyroidism, with an annual incidence of 2.6% in the absence of anti-TPO and 4.3% in its presence.

Limitations

The study had various constraints, such as a limited sample size and a single-center design, which could restrict the applicability of the results. The study's cross-sectional design precludes the evaluation of temporal changes or causality. The study population was restricted to individuals who were diagnosed with autoimmune connective tissue disorders and who tested positive for ANA. It is possible that the study did not properly account for regional differences or variances in ethnicity. The presence of exclusion criteria could have caused a selection bias, while the dependence on certain diagnostic techniques may impact the accuracy and reproducibility of the findings. The absence of longitudinal data hinders the comprehension of the development of thyroid dysfunction in autoimmune CTDs, and the similarity of symptoms could confound the process of diagnosing and evaluating the condition.

## Conclusions

This study underscores the significant prevalence of thyroid dysfunction among patients with ANA-positive autoimmune CTDs. Thyroid abnormalities, including subclinical hypothyroidism, hypothyroidism, and hyperthyroidism, were prevalent, especially among female participants. The findings highlight the necessity for routine thyroid function screening in patients with autoimmune CTDs, as early detection and management of thyroid abnormalities can prevent the progression to more severe thyroid disorders and improve overall patient outcomes. This proactive approach is crucial for mitigating the impact of thyroid dysfunction on the quality of life of these patients.

Additionally, the presence of thyroid autoantibodies, particularly anti-TPO antibodies, further reinforces the strong association between thyroid dysfunction and autoimmune CTDs. The study indicates that autoimmune diseases often coexist and share similar pathophysiological mechanisms. This highlights the need for a holistic approach to patient care, where clinicians not only address the primary autoimmune condition but also monitor and manage associated thyroid dysfunctions. By adopting comprehensive management plans that consider the interconnected nature of these conditions, healthcare providers can ensure more effective treatment strategies and enhance the overall well-being of patients with autoimmune CTDs.
